# Risk factors for bloodstream infection in COVID-19 patients in intensive care units: a systematic review and meta-analysis

**DOI:** 10.1186/s12879-024-10420-1

**Published:** 2025-01-03

**Authors:** Jun Wang, Ting Jiang

**Affiliations:** 1 Laboratory Department, First Teaching Hospital of Tianjin University of Traditional Chinese Medicine, Tianjin, China; 2 Intensive Care Unit, First Teaching Hospital of Tianjin University of Traditional Chinese Medicine, Tianjin, China; 3 National Clinical Research Center for Chinese Medicine Acupuncture and Moxibustion, Tianjin, 300193 China

**Keywords:** Bloodstream infection, COVID-19, ICU, Meta-analysis

## Abstract

**Background:**

Risk factors for bloodstream infection in patients with COVID-19 in the intensive care unit (ICU) remain unclear. The purpose of this systematic review was to study the risk factors for BSI in patients admitted to ICUs for COVID-19.

**Methods:**

A systematic search was performed on PubMed, EMBASE, Cochrane Library, and Web of Science up to July 2024. Data were reported as combined odds ratio (OR) for categorical variables and weighted mean difference (WMD) for continuous variables.

**Results:**

6914 studies were retrieved, of which 55 were included in the meta-analysis. Men (OR = 1.28, 95% CI: 1.10–1.50, *P* = 0.006), high SAPS II score (WMD = 6.43, 95% CI: 0.23–12.63, *P* = 0.042), diabetes (OR = 1.34, 95% CI: 1.04–1.73, *P* = 0.022), tracheal intubation (OR = 8.68, 95% CI: 4.68–16.08, *P* < 0.001), mechanical ventilation (OR = 22.00, 95% CI: 3.77-128.328, *P* < 0.001), ECMO (OR = 2.70, 95% CI: 1.17–6.26, *P* = 0.020), central venous cannulation (OR = 9.33, 95% CI: 3.06–28.43, *P* < 0.001), prolonged ICU stay (WMD = 10.37, 95% CI: 9.29–11.44, *P* < 0.001), methylprednisolone use (OR = 2.24, 95% CI: 1.24–4.04, *P* = 0.008), and the combination of methylprednisolone and Tocilizumab (OR = 4.54, 95% CI: 1.09–18.88, *P* = 0.037) were risk factors for ICU-BSI in COVID-19 patients.

**Conclusion:**

We identified 10 risk factors for ICU-BSI in COVID-19 patients. In future studies, these factors can be combined to establish a more comprehensive and accurate prediction model for ICU-BSI in COVID-19 patients. Targeted measures can be taken earlier to control BSI.

**Supplementary Information:**

The online version contains supplementary material available at 10.1186/s12879-024-10420-1.

## Background

COVID-19 has had a severe impact on global public health systems and remains one of the most severe epidemics now over the world. As of January 2023, WHO reported about 750 million confirmed cases of COVID-19 globally, covering about 6.8 million deaths [1]. The virus is constantly mutating, and the number of infected people continues to grow rapidly. According to statistics from outpatient and emergency clinics in various hospitals, about 40–50% of the patients are prone to serious illnesses and thus require hospital admission. Of these, 10–20% of patients are prone to critical illnesses and need to be admitted to intensive care units (ICUs) for supportive treatment. Consequently, the number of COVID-19 patients admitted to ICUs has markedly increased, and in some places, temporary ICUs have even been built in large numbers. Previous studies have shown that bloodstream infection (BSI) may occur in approximately 7% of COVID-19 inpatients [2]. The incidence of BSI in COVID-19 patients in ICUs is 10–50%, which is a significant increase and a new record and is four times higher than that in non-COVID-19 patients [3]. BSI can lead to bacterial or viral infections in various organs. In poorly treated or severe cases, it may lead to sepsis, resulting in fever and generalized pain, which gravely impacts the health. In severe cases, it can lead to the risk of death. ICU-BSI increases the risk of 30-day mortality by 40% [4]. In addition, resuscitation therapy such as mechanical ventilation and endotracheal intubation in ICU increases the risk of BSI and poses a great challenge to anti-infective treatment [5–7]. COVID-19 infection may become a small-scale recurrent epidemic pattern in the future. Thereby, it is necessary to stratify patients at risk of BSI and take timely measures to reduce the occurrence of BSI.

Recent studies found that gender, SAPS II score, underlying medical complications [e.g., diabetes mellitus (DM), hypertension], treatment-related factors (e.g., mechanical ventilation, intubation, ECMO), and drug-related factors (e.g., Tocilizumab, Methylprednisolone) might be associated with an increased risk of BSI in critically ill patients with COVID-19 in ICUs. However, the conclusions of various studies about the risk factors for BSI are inconsistent. Regarding gender, most studies found that men had a higher risk of developing BSI, which reached 60–70%, and men accounted for most COVID-19 admissions to ICUs [8–13]. Some studies concluded that gender was not associated with the risk of infection [14–18]. Interestingly, 2 studies found the risk of BSI was equal for males and females [19, 20]. DM, as the most common underlying disease in human beings, is statistically associated with a higher risk of BSI in most studies [8, 10, 13, 15–18, 20, 21]. However, other studies concluded that DM did not lead to a higher risk of BSI [9, 11, 12]. Additionally, the conclusions regarding hypertension were not clear and consistent. In most studies, statistical analyses suggested that hypertension caused a higher risk of BSI [10–13, 16, 18, 19]; whereas some studies indicated no direct correlation between hypertension and the risk of BSI [8, 17, 20]. Therefore, in this study, we aimed to determine the risk factors for BSI in COVID-19 patients in ICUs through a systematic review and meta-analysis.

## Methods

This paper was designed and revised following the Preferred Reporting Items for Systematic Reviews and Meta-Analyses and was registered in the PROSPERO (CRD42023416813).

### Search strategy

PubMed, Embase, Web of Science, and Cochrane Library were comprehensively searched for English papers up to July 2024, while references in related literature were manually checked. The following keywords were utilized to screen published clinical information on risk factors for BSI in COVID-19 patients in ICUs: “intensive care unit”, “covid-19”, “sepsis”, and “bloodstream infection”. All literature retrieved was imported into EndnoteX9 for paper screening. The search process was undertaken by two reviewers and any disagreements were addressed through discussion. (Table S1)

### Eligibility criteria

Inclusion criteria covered: (1) study type: observational study; (2) patients: with COVID-19 in ICUs; (3) study content: risk factors for BSI in COVID-19 patients in ICUs. Exclusion criteria encompassed: (1) letters, reviews, conference proceedings, commentaries, and papers with unavailable full text and unsuitable types of publication; (2) with no available primary data; (3) published not in English.

### Data extraction

Based on the eligibility criteria, articles imported into EndnoteX9 were initially screened by reviewing the titles and abstracts. Ineligible articles were excluded, and the remaining articles were read through the full text to screen the eligible ones for meta-analysis. Relevant research data were extracted. All procedures were performed by two researchers (Ting Jiang and Jun Wang). The following data were extracted: (1) general information: authors, publication date, study area, study design, and period; (2) study characteristics: sample size, mean age, and gender distribution; (3) risk factors: SAPS II, hypertension, DM, chronic pulmonary disease, liver disease, immunosuppression, chronic kidney disease, heart disease, and tumors; and (4) treatment records: medication administration, treatment modalities, and duration of treatment. In studies where some information was lacking, we attempted to contact the authors by phone or email. In case of disagreement in literature screening and data extraction, a third researcher (Wei Wang) was consulted.

### Quality assessment

Two researchers independently assessed the study quality based on the Newcastle-Ottawa Scale (NOS) for cohort studies and case-control studies. The NOS scale covered three dimensions and eight items, with a maximum score of 9 points. A score < 4 was defined as low quality, 4–6 as moderate quality, and ≥ 7 as high quality. The higher score implied a lower risk of bias. The quality of cross-sectional studies was evaluated using a scale recommended by the Agency for Healthcare Research and Quality. The scale consisted of 11 items. Answers included yes, no, and unclear. For the answer of “yes”, the item was assigned a score of 1. The higher the score, the higher the quality: low quality = 0–3; medium quality = 4–7; high quality = 8–11. If two researchers disagreed with quality assessment, a third researcher arbitrated.

### Statistical analyses

Statistical analyses were implemented using Stata 15.0 software. Categorical variables were analyzed using odds ratio (OR) while continuous variables using weighted mean difference (WMD). Data from the original studies were transformed before meta-analysis if they were not described as mean and standard deviation. All effect sizes were represented as 95% confidence intervals (CI). Heterogeneity was analyzed using I^2^. If there was no significant statistical heterogeneity between outcomes (*P* ≥ 0.1, I^2^ ≤ 50%), a meta-analysis was performed using a fixed-effects model, otherwise, using a random-effects model. For highly heterogeneous results, sensitivity analyses were performed on the results to validate the stability and reliability of the results. Sensitivity analyses were conducted on all outcomes. By eliminating the articles one by one, the stability of the remaining results was observed. For risk factors that included ≥ 10 articles, the Egger test was adopted to determine whether there was publication bias.

## Results

### Screening results

**Fig. 1:**
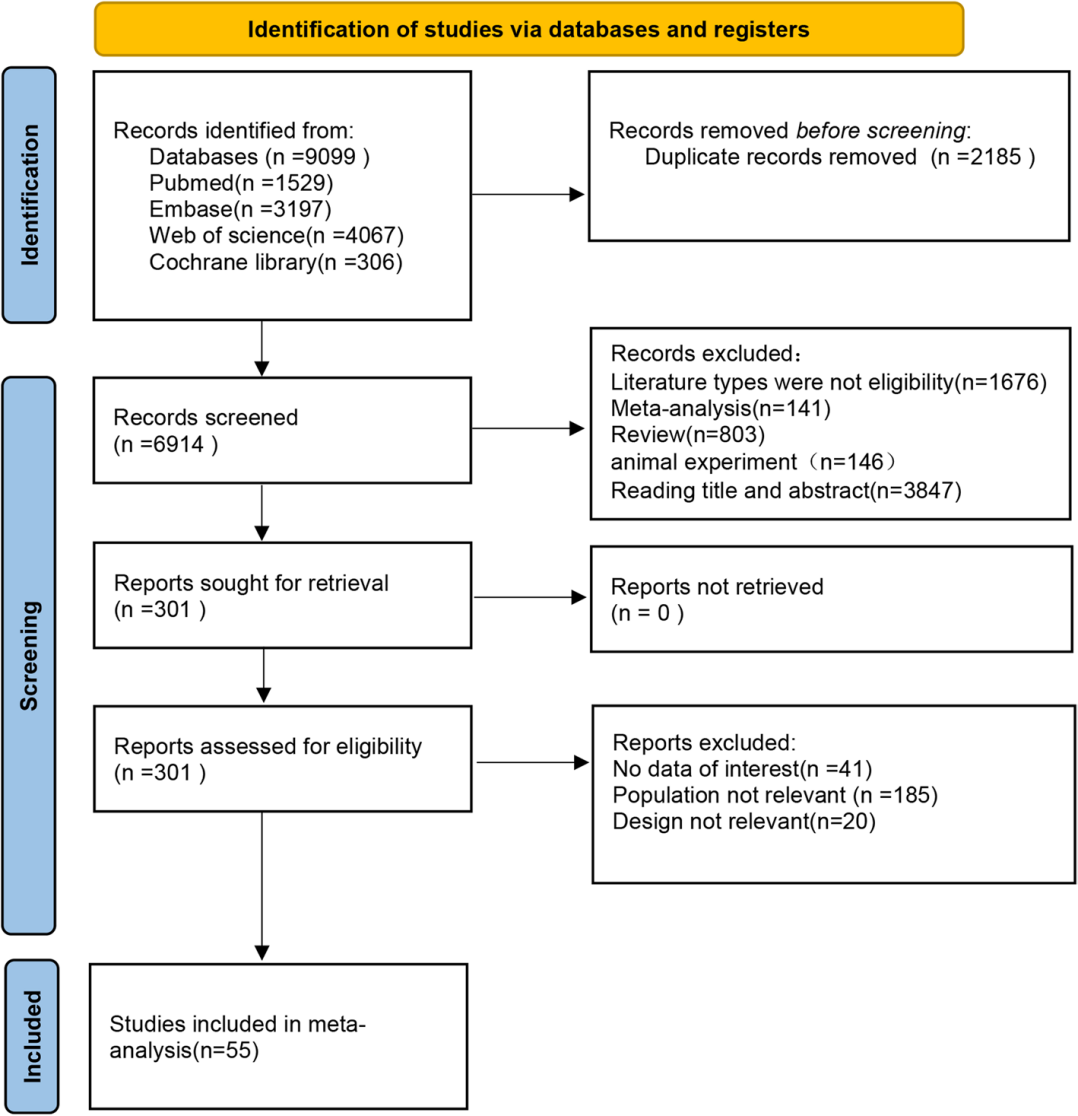
Flowchart of the search strategy

The database searches retrieved 9099 relevant English articles. 6914 articles were obtained after duplicates were removed. Subsequently, the titles and abstracts were read to exclude the ineligible studies, leaving 301 studies. Finally, after reading the full text, 55 studies were enrolled in the meta-analysis [3, 5–58]. The screening process is represented in a PRISMA flowchart. (Fig. 1).

### Characteristics

The 55 included studies comprised 48 cross-sectional studies [5–9, 11, 12, 14, 15, 17, 18, 20–23, 26–58] (Table 1), 6 cohort studies [3, 10, 13, 16, 24, 25], and 1 case-control study [19] (Table 2), including 25,939 patients ranging in age from 18 to 94 years across Italy, CHN, UK, US, Spain, France, Greece, Austria, Singapore, India, Germany, Turkey, Switzerland, Sweden, and Portugal. The meta-analysis results of the research indicators are shown in Table 3. Among them, 48 cross-sectional studies had average AHRQ scale scores > 7, and 6 cohort studies and 1 case-control study had average NOS scores > 7, implying that these articles were all of high quality.

**Table 1:** Studies characteristics and quality. (cross-sectional study)

Study	Published time	Country	Study design	Patients	Age	Gender(male)	AHRQ grade
Amit, M. et al. [22]	2020	Israel	Cross-sectional study	156	72(60–82)	108/48	9
Bonazzetti, C et al. [14]	2020	Italy	Cross-sectional study	89	61.5(53.1–68.7)	69/20	8
Fu, Guoping et al. [23]	2020	CHN	Cross-sectional study	51	60.94 ± 14.87(25–87)	27/14	8
Giacobbe, D. R et al. [8]	2020	Italy	Cross-sectional study	78	66 IQR 57–70	60/18	9
Anwar, Asad et al. [26]	2021	UK	Cross-sectional study	44	17–77 M59.5(IQR 50.5–64.5)/21–80 M59(IQR 49–67.5)	34/10	8
Bardi, Tommaso et al. [27]	2021	Spain	Cross-sectional study	140	61(57–67)	108/32	8
d’Humières, C et al. [15]	2021	FRANCE	Cross-sectional study	197	59(50–68)	148/49	8
Dupuis, Claire et al. [28]	2021	FRANCE	Cross-sectional study	303	61(53–70)	239/64	9
Grasselli, G. et al. [29]	2021	Italy	Cross-sectional study	774	62 (54–68)	597/177	9
Karruli, A. et al. [9]	2021	Italy	Cross-sectional study	32	68 [55.25–75]	23/9	9
Kokkoris, S. et al. [30]	2021	Greece	Cross-sectional study	50	Median age 64	36/14	8
Llitjos, Jean-Francois et al. [5]	2021	FRANCE	Cross-sectional study	176	63 (55–73)	134/42	8
Ong, C. C. H. et al. [31]	2021	Singapore	Cross-sectional study	71	52(39–66)	59/12	9
Ramos, Rafael et al. [32]	2021	Spain	Cross-sectional study	213	61(52–71)	110/103	8
Roedl, Kevin et al. [33]	2021	Germany	Cross-sectional study	223	69 (58–77.5)	163/60	8
Rollas, Kazim et al. [17]	2021	Turkey	Cross-sectional study	38	NR	NR	9
Søgaard, K. K et al. [34]	2021	Switzerland	Cross-sectional study	41	64.8(54.7–72.1)	31/10	9
Suarez-de-la-Rica, A. et al. [35]	2021	Spain	Cross-sectional study	107	62.2 ± 10.6	76/31	8
Yakar, Mehmet Nuri et al. [36]	2021	Turkey	Cross-sectional study	249	71(61–80)	172/77	9
Yao, Ren-qi et al. [6]	2021	CHN	Cross-sectional study	35	64(59–67)	25/10	8
Zamora-Cintas, M. I. et al. [37]	2021	Spain	Cross-sectional study	54	NR	NR	8
Zhang, J. et al. [18]	2021	CHN	Cross-sectional study	32	63.34 ± 12.48	20/12	9
Ahlstrom, Bjorn et al. [38]	2022	Swedish	Cross-sectional study	7382	63 (53–72)	5191/2191	9
Brücker, W. et al. [39]	2022	Germany	Cross-sectional study	61	66.4 ± 13.3	34/27	9
Caiazzo, L.et al. [40]	2022	Italy	Cross-sectional study	89	68.1 ± 9.3	66/23	8
Cidade, Jose Pedro et al. [41]	2022	Portugal	Cross-sectional study	118	63.3 ± 13.1	87/31	8
Ćurčić, M. et al. [42]	2022	Croatia	Cross-sectional study	692	NR	NR	8
da Costa, R. L. et al. [43]	2022	Brazil	Cross-sectional study	191	69.66 ± 16.13	116/75	8
De Bruyn, A. et al. [44]	2022	Belgium.	Cross-sectional study	94	69.65 ± 11.29	55/39	9
DeVoe, C. et al. [45]	2022	US	Cross-sectional study	126	58.1 ± 17.9	85/41	8
Erbay, Kubra et al. [11]	2022	Turkey	Cross-sectional study	85	67.23 ± 13.05	54/31	8
Kozlowski, Bartosz et al. [46]	2022	Poland	Cross-sectional study	172	67.76 ± 11.16	112/60	8
Kurt, Ahmet Furkan et al. [12]	2022	Turkey	Cross-sectional study	470	66 ± 14.87	301/169	9
Lepape, Alain et al. [47]	2022	FRANCE	Cross-sectional study	4465	63.30 ± 11.68	3132/1333	8
Mantzarlis, K. et al. [20]	2022	Greece	Cross-sectional study	84	68.85 ± 12.17	56/28	9
Mustafa, Z. U. et al. [48]	2022	Pakistan	Cross-sectional study	636	NR	398/238	7
Pandey, M. et al. [49]	2022	UK	Cross-sectional study	299	NR	101/198	9
Roda, Silvia et al. [50]	2022	Italy	Cross-sectional study	22	61.36 ± 10.30	20/2	8
Routsi, C. et al. [51]	2022	Greece	Cross-sectional study	600	NR	NR	8
Russo, A. et al. [52]	2022	Italy	Cross-sectional study	32	62.50 ± 10.99	21/11	8
Seitz, T. et al. [53]	2022	Austria	Cross-sectional study	117	57.2 ± 11.9	72/45	8
Torrecillas, Miriam et al. [7]	2022	Spain	Cross-sectional study	220	63.65 ± 12.69	169/51	8
Alenazi, T. A. et al. [54]	2023	Saudi Arabia	Cross-sectional study	118	60.97 ± 16.32	74/43	8
Alessandri, F. et al. [55]	2023	Italy	Cross-sectional study	138	62.20 ± 15.36	97/41	9
Bedenić, B. et al. [56]	2023	Croatia	Cross-sectional study	118	71 years (range 25–94)	78/40	8
Bonazzetti, C. t al. [21]	2023	Italy	Cross-sectional study	537	64.65 ± 11.15	402/135	9
Guanche Garcell, H. et al. [57]	2023	Cuban	Cross-sectional study	130	NR	NR	8
Taysi, M. R. et al. [58]	2023	Turkey	Cross-sectional study	205	68.4 ± 13.1	119/86	8

*AHRQ* Agency for Healthcare Research and Quality, *NR* Not reported

**Table 2:** Studies characteristics and quality. (Cohort study and case-control study)

Study	Published time	Country	Study design	Patients	Age	Gender (male)	NOS grade
Cataldo, M. A et al. [3]	2020	Italy	Cohort study	57	62 ± 13	41/16	7
Garcia, Pedro David Wendel et al. [24]	2020	European	Cohort study	639	63 (53–71)	480/159	8
Zhang, H. et al. [25]	2020	CHN	Cohort study	38	64.76 ± 13.76	32/6	7
Massart, N. et al. [10]	2021	France, Switzerland, Belgium	Cohort study	4010	NR	NR	8
Palanisamy, N. et al. [16]	2021	India	Cohort study	750	60 ± 17.71	562/188	8
Bartoszewicz, M. et al. [13]	2023	Poland	Cohort study	201	66.1 ± 12.1	114/87	8
Dupper, A. C. et al. [19]	2022	US	Case-control study	96	64.91 ± 9.51	57/39	8

*NOS *Newcastle-Ottawa Scale, *NR* Not reported

**Table 3:** Outcomes of meta-analysis

Risk factors	No. of studies	Heterogeneity Analysis		Statistical model	statistical method	Effect estimate	*P*
		I²	*P*			(95%CI)	
Hypertension	10	70.4%	0.000	Random-effects	OR	1.30(0.92,1.83)	0.131
Chronic pulmonary disease	11	23.4%	0.221	Fixed-effects	OR	1.07(0.90,1.29)	0.443
Diabetes	12	50.2%	0.024	Random-effects	OR	1.34(1.04,1.73)	0.022*
Gender	14	0.0%	0.059	Fixed-effects	OR	1.28(1.10,1.50)	0.006*
Liver disease	6	2.3%	0.402	Fixed-effects	OR	0.86(0.47,1.58)	0.635
Immunosuppressive diseases	5	29.9%	0.222	Fixed-effects	OR	1.11(0.88,1.40)	0.375
Chronic kidney disease	6	0.0%	0.751	Fixed-effects	OR	1.20(0.78,1.84)	0.411
Heart disease	10	0.0%	0.550	Fixed-effects	OR	1.00(0.85,1.17)	0.957
Tocilizumab	9	34.3%	0.144	Fixed-effects	OR	1.04(0.74,1.46)	0.815
Tumors	9	10.2%	0.350	Fixed-effects	OR	1.04(0.78,1.37)	0.807
ECMO	4	74.1%	0.009	Random-effects	OR	2.70(1.17,6.26)	0.020*
Tracheal intubation	4	67.8%	0.025	Random-effects	OR	8.68(4.68,16.08)	< 0.001*
Mechanical ventilation	2	0.0%	0.385	Fixed-effects	OR	22.00(3.77,128.328)	0.001*
Methylprednisolone	2	13.5%	0.282	Fixed-effects	OR	2.24(1.24,4.04)	0.008*
Methylprednisolone + Tocilizumab	2	71.0%	0.063	Random-effects	OR	4.54(1.09,18.88)	0.037*
Steroids	3	87.6%	0.000	Random-effects	OR	1.17(0.15,9.23)	0.882
Remdesivir	2	54.4%	0.139	Random-effects	OR	0.80(0.14,4.41)	0.794
Dexamethasone	2	10.2%	0.291	Fixed-effects	OR	1.64(0.85,3.15)	0.139
Renal replacement therapy	2	97.9%	0.000	Random-effects	OR	0.86(0.11,6.57)	0.882
Central venous catheterization	2	0.0%	0.559	Fixed-effects	OR	9.33(3.06,28.43)	< 0.001*
Length of stay in ICUs	8	0.0%	0.712	Fixed-effects	WMD	10.37(9.29,11.44)	< 0.001*
SAPS II score	2	58.3%	0.122	Random-effects	WMD	6.43(0.23,12.63)	0.042*

*WMD* Weight mean difference, *OR* Odds ratio, *CI* Confidence interval

*P < 0.05

### Result synthesis

#### Patient-related factors

##### Gender

**Fig. 2:**
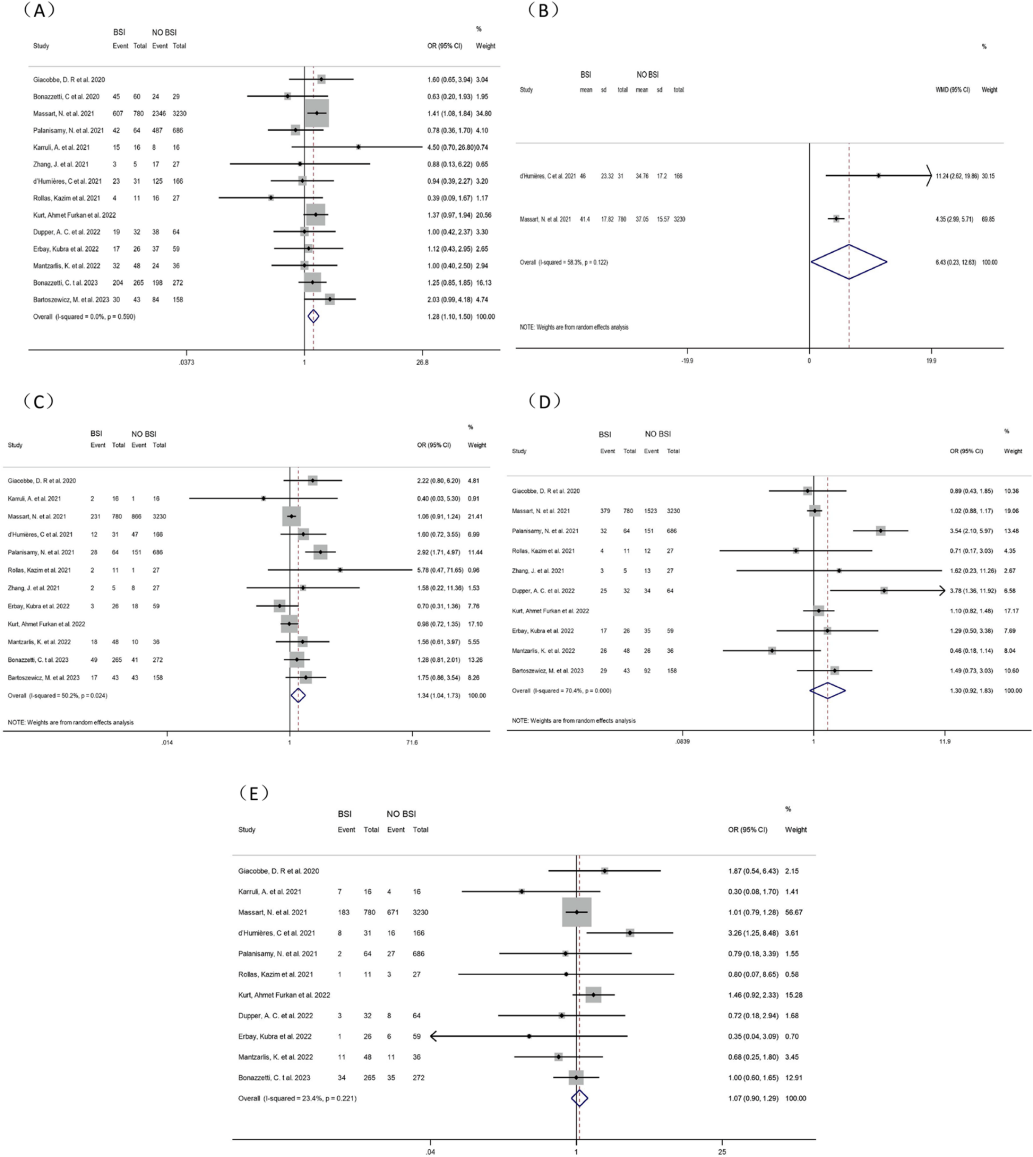
Forest plot of univariate data associating BSI risk with (**A**) gender; (**B**) SAPS II score; (**C**) diabetes; (**D**) hypertension and (**E**) chronic pulmonary disease for patients with COVID-19 in ICU

Fourteen studies [8–21] explored the correlation between gender and BSI in COVID-19 patients in ICUs. A pooled analysis showed that male COVID-19 patients in ICUs were 28% more likely to develop BSI (OR = 1.28, 95% CI: 1.10–1.50, *P* = 0.006, I^2^ = 0.0%). (Fig. 2)

##### SAPS II score

Two studies [10, 15] analyzed the correlation between SAPS II scores and ICU-BSI in COVID-19 patients. Meta-analysis showed that higher SAPS II scores were positively correlated with an increased incidence of BSI in COVID-19 patients in ICUs (WMD = 6.43, 95% CI: 0.23–12.63, *P* = 0.042, I^2^ = 58.3%). (Fig. 2)

##### DM

Twelve studies [8–13, 15–18, 20, 21] investigated the correlation between DM and BSI in COVID-19 patients in ICUs. Most of these articles did not indicate whether DM was a risk factor for BSI. Our pooled analysis unraveled that DM increased the occurrence of BSI in COVID-19 patients in ICUs by 34% (OR = 1.34, 95% CI: 1.04–1.73, *P* = 0.022, I^2^ = 50.2%). (Fig. 2)

##### Hypertension

There were conflicting results about the association between hypertension and BSI in COVID-19 patients in ICUs. Ten studies [8, 10–13, 16–20] were involved with mixed results. Meta-analysis demonstrated no correlation between hypertension and BSI in COVID-19 patients in ICUs (OR = 1.30, 95% CI:0.92–1.83, *P* = 0.131, I^2^ = 70.4%). (Fig. 2)

##### Chronic pulmonary disease

Because COVID-19 mainly attacked the respiratory system, we extensively investigated the correlation between chronic pulmonary disease and BSI in COVID-19 patients in ICUs through 11 studies [8–12, 15–17, 19–21]. Meta-analysis showed no correlation between chronic pulmonary disease and BSI in COVID-19 patients in ICUs (OR = 1.07, 95% CI: 0.90–1.29, *P* = 0.443, I^2^ = 23.4%). (Fig. 2)

##### Liver disease

**Fig. 3:**
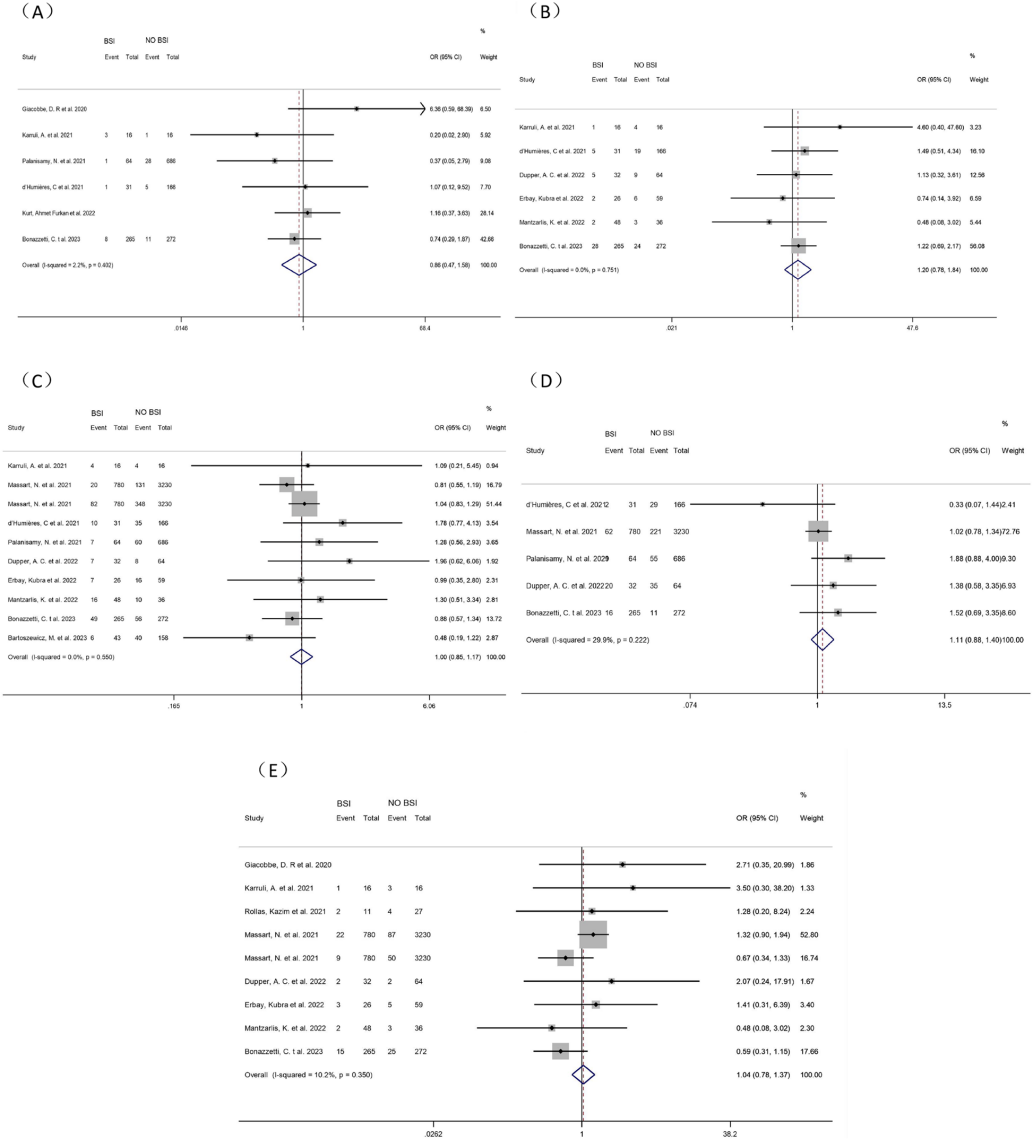
Forest plot of univariate data associating BSI risk with (**A**) liver disease; (**B**) chronic kidney disease; (**C**) heart disease; (**D**) immunosuppressive disease and (**E**) tumors for patients with COVID-19 in ICU

Six studies [8, 9, 12, 15, 16, 21] investigated the correlation between liver disease and ICU-BSI in COVID-19 patients. Meta-analysis showed no correlation between liver disease and BSI in COVID-19 patients in ICUs (OR = 0.86, 95% CI: 0.47–1.58, *P* = 0.635, I^2^ = 2.25%). (Fig. 3)

##### Chronic kidney disease

Seven studies [9–11, 15, 19–21] investigating the association between chronic kidney disease and BSI in COVID-19 patients in ICUs were included. One article was excluded by sensitivity analysis [10] and therefore six articles were included in the meta-analysis. It showed no correlation between chronic kidney disease and BSI in COVID-19 patients in ICUs (OR = 1.20, 95% CI: 0.78–1.84, *P* = 0.411, I^2^ = 0.0%). (Fig. 3)

##### Heart disease

Ten studies [9–11, 13, 15, 16, 19–21] investigating the correlation between heart disease and BSI among COVID-19 patients in ICUs were included. Meta-analysis showed no correlation between heart disease and BSI in COVID-19 patients in ICUs (OR = 1.00, 95% CI: 0.85–1.17P = 0.957, I^2^ = 0.0%). (Fig. 3)

##### Immunosuppressive diseases

All five studies [9, 15, 16, 19, 21] showed no correlation between immunosuppression and ICU-BSI in COVID-19 patients. Meta-analysis also showed no correlation between immunosuppression and BSI in COVID-19 patients in ICUs (OR = 1.11, 95% CI: 0.88–1.40, *P* = 0.375, I^2^ = 29.9%). (Fig. 3)

##### Tumors

Nine studies [8–11, 17, 19–21] investigating the correlation between tumors and BSI in COVID-19 patients in ICUs were included. Meta-analysis showed no correlation between tumors and BSI in COVID-19 patients in ICUs (OR = 1.04, 95% CI: 0.78–1.37, *P* = 0.807, I^2^ = 10.2%). (Fig. 3)

#### Treatment-related factors

##### Tracheal intubation

**Fig. 4:**
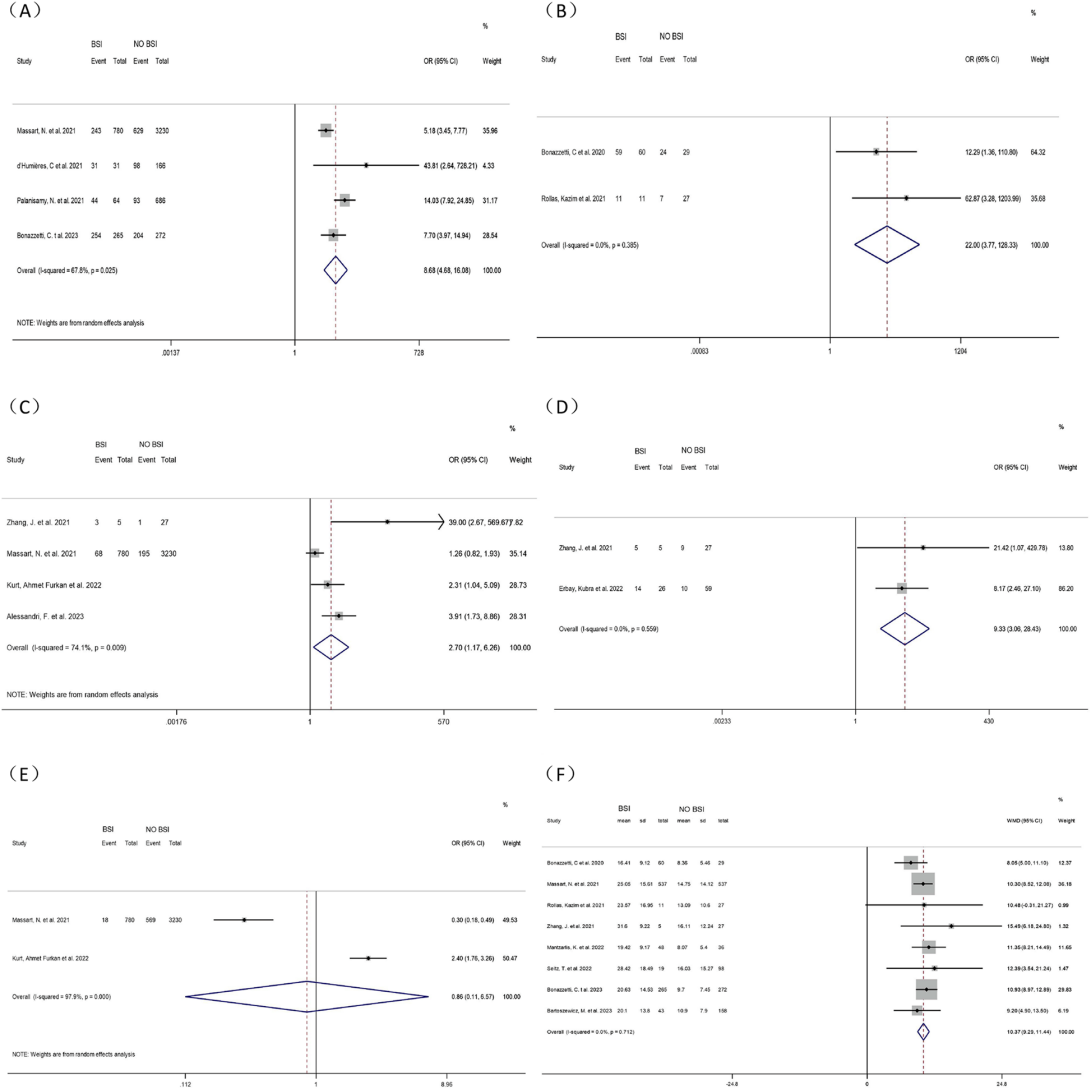
Forest plot of univariate data associating BSI risk with (**A**) tracheal intubation; (**B**) mechanical ventilation; (**C**) ECMO; (**D**) CVC; (**E**) RRT and (**F**) Length of stay in ICU for patients with COVID-19 in ICU

Four studies [10, 15, 16, 21] were included to investigate the association between tracheal intubation and ICU-BSI in COVID-19 patients. Meta-analysis revealed that tracheal intubation increased the risk of BSI in COVID-19 patients in ICUs by nearly 9-fold (OR = 8.68, 95% CI: 4.68–16.08, *P* < 0.001, I^2^ = 67.8%). (Fig. 4)

##### Mechanical ventilation

The correlation between mechanical ventilation and BSI in COVID-19 patients in ICUs was investigated by three studies [14, 16, 17]. Since no heterogeneity was found (*P* = 0.147, I^2^ = 47.9%), a fixed-effects model was adopted and unraveled marked differences (OR = 4.98, 95% CI: 2.73–9.08, *P* < 0.001). After sensitivity analysis, the heterogeneity was greatly reduced (*P* = 0.385, I^2^ = 0.0%) when the study of Palanisamy, N et al. [16] was excluded. The main source of heterogeneity might be the large sample size of their study, which tended to lead to unstable results compared to other studies with small sample sizes. Thus, this study was excluded because it led to a significant bias. The pooled analysis after exclusion using a fixed-effect model (OR = 22.00, 95% CI: 3.77–128.328, *p* < 0.001) showed statistically significant differences. The meta-analysis showcased that mechanical ventilation increased the risk of BSI by 22 times in COVID-19 patients in ICUs. The excluded study by Palanisamy, N et al. also showed that mechanical ventilation could increase the risk of BSI by 4-fold, in agreement with our results. (Fig. 4)

##### ECMO

Many critically ill patients have used ECMO for supportive care. Including four studies [10, 12, 18, 55], we explored the correlation between ECMO and BSI among COVID-19 patients in ICUs. Meta-analysis manifested that ECMO increased the risk of BSI in COVID-19 patients in ICUs by nearly three times (OR = 2.70, 95% CI: 1.17–6.26, *P* = 0.020, I^2^ = 74.1%). (Fig. 4)

##### Central venous catheterization (CVC)

Two studies [11, 18] investigated the correlation between CVC and ICU-BSI in COVID-19 patients. Meta-analysis showed that CVC increased the Catheter-related BSI (OR = 9.33, 95% CI: 3.06–28.43, *P* < 0.001, I^2^ = 0.0%). (Fig. 4)

##### Renal replacement therapy (RRT)

Two studies [10, 12] investigating the correlation between RRT and BSI in COVID-19 patients in the ICU were included. Meta-analysis showed no correlation between BSI and RRT in COVID-19 patients in ICUs (OR = 0.86, 95% CI: 0.11–6.57, *P* = 0.882, I^2^ = 97.9%). (Fig. 4)

##### Length of stay in ICUs

Eight studies were included [12–14, 17, 18, 20, 21, 53], all of which showed a strong correlation between the length of stay in ICUs and the occurrence of BSI in COVID-19 patients in ICUs. A meta-analysis showed that the longer the ICU stay, the higher the risk of BSI in COVID-19 patients in the ICU (WMD = 10.37, 95% CI:9.29–11.44, *P* < 0.001, I^2^ = 0.0%). (Fig. 4)

#### Medication-related factors

##### Tocilizumab

**Fig. 5:**
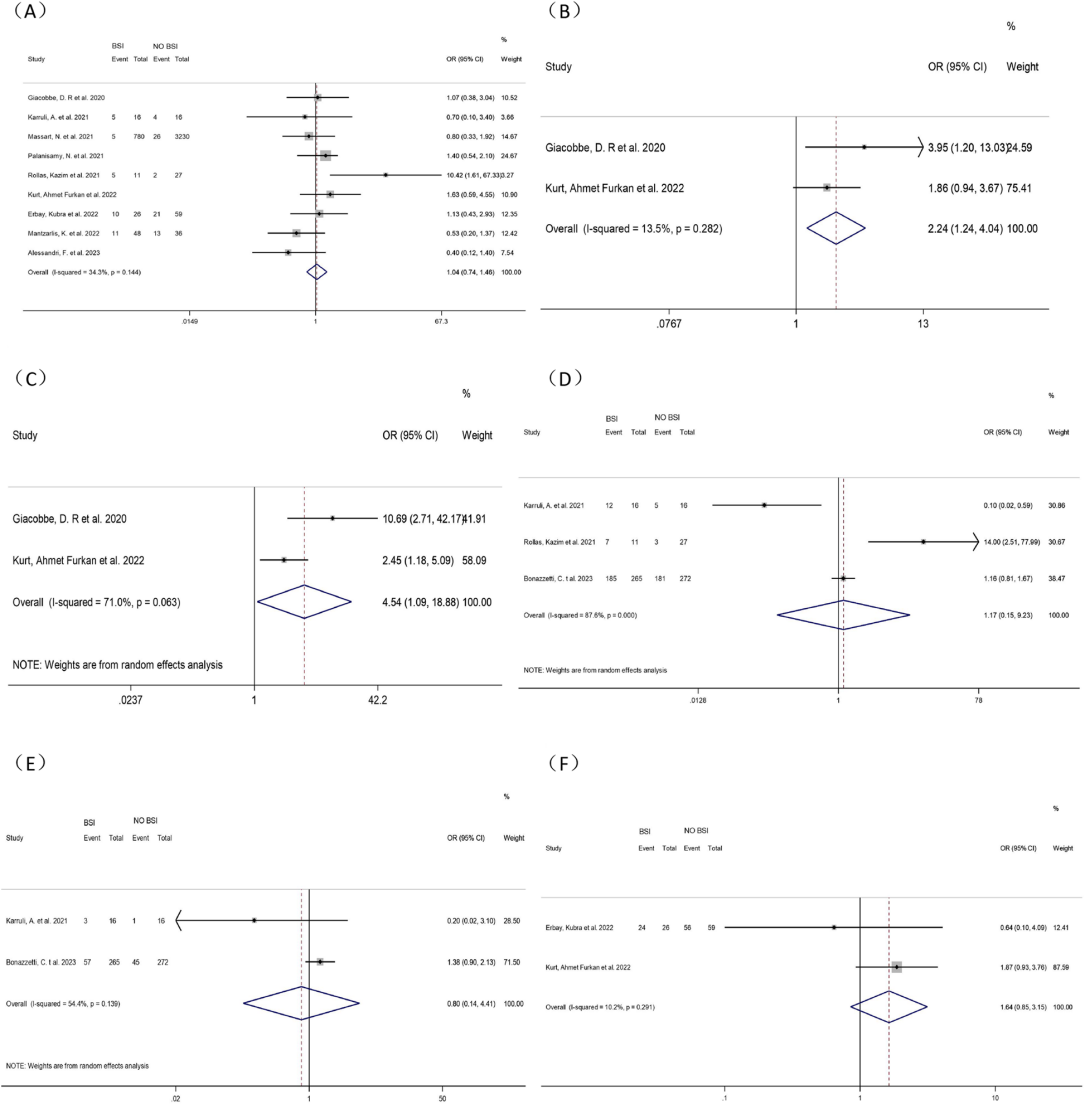
Forest plot of univariate data associating BSI risk with (**A**) Tocilizumab; (**B**) Methylprednisolone; (**C**) Methylprednisolone and Tocilizumab combination; (**D**) Steroids; (**E**) Remdesivir and (**F**) Dexamethasone for patients with COVID-19 in ICU

The correlation between Tocilizumab and BSI in COVID-19 patients was investigated in 10 studies [8–12, 16, 17, 20, 21, 55]. One article was excluded by sensitivity analysis [21] and therefore nine articles were enrolled in the meta-analysis. There was no correlation between Tocilizumab and BSI in COVID-19 patients in ICUs (OR = 1.04, 95% CI: 0.74–1.46, *P* = 0.815, I^2^ = 34.3%). However, this may explain why Tocilizumab is widely used for severe and critically ill COVID-19 patients in ICUs under the guidance of guidelines. (Fig. 5)

##### Methylprednisolone

Two studies [8, 12] investigated the association between Methylprednisolone and ICU-BSI in COVID-19 patients. Meta-analysis signified that Methylprednisolone was linked with BSI in COVID-19 patients in ICUs (OR = 2.24, 95% CI: 1.24–4.04, *P* = 0.008, I^2^ = 13.5%). Meanwhile, we found that the combination of Methylprednisolone and Tocilizumab significantly increased the risk for BSI in COVID-19 patients in ICUs (OR = 4.54, 95% CI: 1.09–18.88, *P* = 0.037, I^2^ = 71%). (Fig. 5)

##### Steroids

All the studies on the risk of steroid use on ICU-BSI in COVID-19 patients were included, and the results were found only in 3 studies [9, 17, 21]. Meta-analysis showed no correlation between steroid use and BSI in COVID-19 patients in ICUs (OR = 1.17, 95% CI: 0.15–9.23, *P* = 0.882, I^2^ = 87.6%). This may be related to the fact that steroids are widely used as they are believed to improve the recovery of patients. (Fig. 5)

##### Dexamethasone

Two studies [11, 12] investigated the association between Dexamethasone and ICU-BSI in COVID-19 patients. Meta-analysis showed no correlation between Dexamethasone use and BSI in COVID-19 patients in ICUs (OR = 1.64, 95% CI: 0.85–3.15, *P* = 0.139, I^2^ = 10.2%) (Fig. 5).

##### Remdesivir

The correlation between Remdesivir and ICU-BSI in COVID-19 patients was investigated in 2 studies [9, 21]. Meta-analysis showed no correlation between Remdesivir and BSI in COVID-19 patients in ICUs (OR = 0.80, 95% CI: 0.14–4.41, *P* = 0.794, I^2^ = 54.4%). This may be related to the fact that Remdesivir is considered a potent drug for the treatment of COVID-19, with significant efficacy, and therefore is more widely used for severe and critically ill patients in ICUs. (Fig. 5)

### Sensitivity analysis

The stability of the results of the remaining articles was estimated by excluding each article in turn. Sensitivity analyses for gender, SAPS II score, DM, hypertension, chronic pulmonary disease, liver disease, heart disease, immunosuppressive disease, tumor, tracheal intubation, ECMO, CVC, RRT, length of stay in ICUs, and the use of Methylprednisolone, Steroids, and Remdesivir revealed that the results were relatively stable. In the sensitivity analysis of mechanical ventilation, the study by Palanisamy, N et al. [16] greatly impacted the results, so the results were pooled after the exclusion of that article, and the results were more stable. Similarly, in the sensitivity study of chronic kidney disease, it was found that Massart, N et al. [10] greatly influenced the results. After excluding the article and re-combining the results, the results were more stable. In the sensitivity study on the use of Tocilizumab, the study by Bonazzetti, C et al. [21] greatly influenced the results. The results were more stable when the article was excluded, and the results were re-combined. The sensitivity analyses of other factors implied stable and insignificant changes, so these studies were retained. (Figs. S1, S2, S3)

### Publication bias

**Fig. 6:**
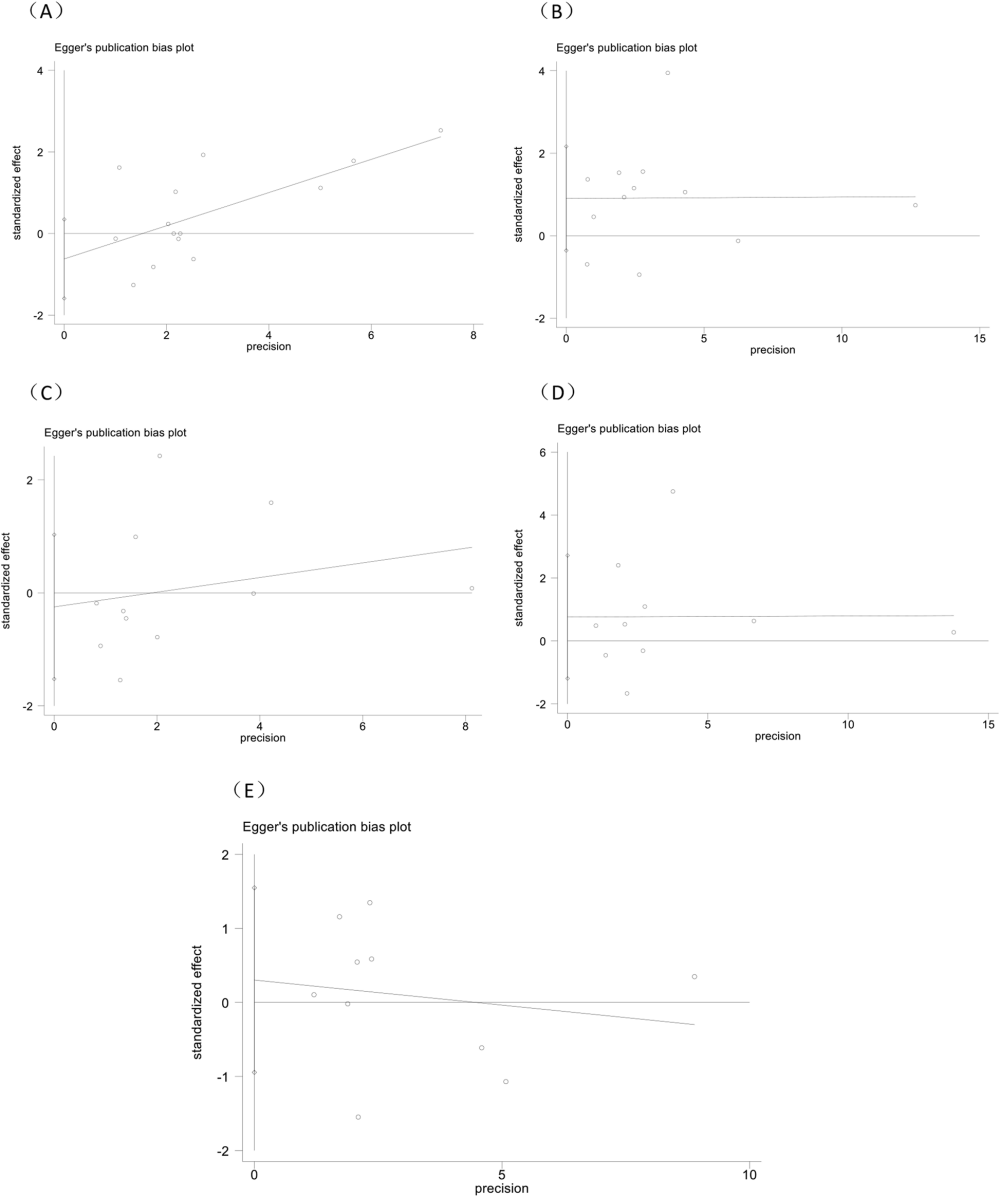
Publication bias of univariate data associating BSI risk with (**A**) gender; (**B**) diabetes; (**C**) chronic pulmonary disease; (**D**) hypertension and (**E**) heart disease for patients with COVID-19 in ICU

Publication bias was examined using the Egger test for the risk factor containing ≥ 10 articles. There was no publication bias for men (*P* = 0.187), DM (*P* = 0.142), hypertension (*P* = 0.396), heart disease (*P* = 0.592), and chronic pulmonary disease (*P* = 0.671). (Fig. 6)

## Discussion

In this meta-analysis, we aimed to identify risk factors for BSI in COVID-19 patients in ICUs. Among the studies with available data, 55 published English studies [3, 5–58] investigating risk factors associated with BSI in COVID-19 patients in ICUs were included. Our findings showed that male, DM, tracheal intubation, mechanical ventilation, CVC, ECMO, Methylprednisolone use, higher SAPS II score, and longer ICU stay were risk factors for ICU-BSI in COVID-19 patients. In addition, hypertension, chronic pulmonary disease, liver disease, chronic kidney disease, heart disease, immunosuppression, tumor, RRT, and the use of Tocilizumab, Steroids, and Remdesivir neither increased nor decreased the risk of ICU-BSI in COVID-19 patients.

### Patient-related factors

Our analyses showed that male COVID-19 patients were at a higher risk of BSI in ICUs and that males also made up most of the ICU admission population. A previous study by Zamora-Cintas, M. et al. [37] highlighted that male patients had a higher risk of BSI, in line with our results. COVID-19 virus infection mainly affects pulmonary function, resulting in more patients with pulmonary dysfunction being admitted to the hospital, and more severe patients need to be admitted to ICUs, which makes them more susceptible to BSI. However, we did not study whether smoking was a risk factor for BSI in COVID-19 patients in ICUs and the proportion of men who smoke., which might require more data support. Our findings also showed that the SAPS II score directly reflected the risk of BSI in COVID-19 patients in ICUs, in agreement with the findings of Massart, N et al. [10]. SAPS II score, as an important evaluation component in ICUs, to some extent, also reflects infection indicators, which is directly related to our study. In short, a higher SAPS II score indicates a more severe condition and a worse prognosis [59]. DM is a common chronic underlying disease in clinical practice. Our results showed that DM was also an associated risk factor for ICU-BSI in COVID-19 patients. This may be related to diverse complications associated with DM and the poor resistance of diabetic patients, which makes them susceptible to a variety of related infections [60] and directly increases the risk of BSI [61].

### Treatment-related factors

Our findings suggested that tracheal intubation substantially increased the risk of ICU-BSI in COVID-19 patients, in support of the findings of Bonazzetti, C et al. [14] and Rollas, Kazim et al. [17]. Tracheal intubation is a common resuscitation technique in ICUs and is essential to save patients in respiratory distress. It ensures that the patient receives an adequate supply of oxygen and provides mechanical ventilation support to maintain normal respiratory function [62]. This makes mechanical ventilation support also a possible risk factor for BSI in COVID-19 patients in ICUs. Invasive treatment is highly likely to cause airway damage, and tracheal intubation may introduce bacteria or other pathogens, increasing the risk of infection in patients. It is also easy for micro-aspiration to occur after tracheal intubation, leading to lung infections [63]. All these directly increase the risk of BSI. COVID-19 has become a specific infection that involves the pathophysiology of the lungs, including endothelial and epithelial changes, pulmonary embolism, and microvascular thrombosis. In addition, secondary infectious injury can cause acute lung injury and prolong mechanical ventilation [5]. Zhang, J et al. showed that multiple invasive treatments were important risk factors for BSI. Early extubation and regular assessment of infection should be done, therefore early anti-infective therapy is important [18]. CVC is widely used in the resuscitation of severe and critically ill patients, which is conducive to the measurement of central venous pressure, long-term medication, and large and rapid rehydration, thus preventing venous damage and repeated puncture. However, a common complication of CVC is deep vein thrombosis, which also leads to the invasion of external bacteria and infection. Patients present with persistent low-grade fever, and the simultaneous presence of bacteria and thrombus can exacerbate the infection [64]. Our study revealed that prolonged CVC substantially increased the risk of ICU-BSI in COVID-19 patients. Therefore, there is a need for timely monitoring of the situation and increased measures for infection control and nursing care for CVC. ECMO serves as an important therapeutic tool to provide continuous extracorporeal respiratory and circulatory function for critically ill patients presenting with cardiopulmonary failure. Some studies showed that infections were highly susceptible to occurring after the use of ECMO, which was related to the fact that patients with low immunity were susceptible to systemic hematogenous infections, thus dramatically increasing the risk of fungal infections [65, 66]. This was in general agreement with the results of our study. Meanwhile, ECMO may result in renal failure in about 50% of patients, which may require RRT [67]. However, our study found that RRT was not a risk factor for ICU-BSI in COVID-19 patients, which may need to be supported by more data. In particular, intuitive data in our study pointed out that the longer the treatment duration in ICUs, the higher the risk of BSI in COVID-19 patients. The possible reasons are as follows: first, the treatment time reflects the severity of the patient’s condition, and a longer ICU stay may mean that the patient’s condition is more critical; second, the longer the treatment time, the higher the chance of nosocomial infections [11], which is closely related to the prolonged use of antibiotics and ward management; third, severe and critically ill patients in ICUs have low resistance and need to be left with various passages during treatment, and most COVID-19 patients have coughing symptoms, which is prone to aerosol dissemination and transmission of infectious disease between the patients, thus greatly increasing the risk of BSI [68].

### Medication-related factors

In our study, the use of Tocilizumab, Remdesivir, Steroids, and Dexamethasone did not correlate with the risk of BSI in COVID-19 patients in ICUs, but the use of Methylprednisolone and the combination of Methylprednisolone and Tocilizumab directly increased the risk of ICU-BSI in COVID-19 patients. This is associated with the fact that patients receiving glucocorticoid therapy are more likely to require ventilatory support, vasopressors, and RRT [28, 52]. Glucocorticoids are widely used and effective drugs during hospitalization, especially in ICU, due to their anti-inflammatory and immunosuppressive effects. Glucocorticoids inhibit the inflammatory chemotaxis of cells and the rate of phagocytosis to reach inflammation sites. In addition, glucocorticoids increase the stability of cells so that cell membranes are less likely to rupture, and cells are less likely to release lysosomal enzymes to phagocytose bacteria to destroy inflammatory foci, thus decreasing the body’s immunity and making it susceptible to viral or bacterial infections [69, 70]. Glucocorticoids will also directly inhibit the body’s immune function, thus inhibiting the body’s fever, so that the fever symptoms are not obvious, which in turn masks the severity of the disease and delays the diagnosis and treatment, leading to further deterioration of the condition [28]. Moreover, glucocorticoids also inhibit mucosal exudation and inflammatory exudation. For patients with respiratory tract infections, glucocorticoids inhibit the exudation of inflammatory secretions, so that patients reduce coughing, which is not conducive to discharging bacterial sputum out of the body through coughing. Additionally, it delays the detection and treatment, thus aggravating the infection and increasing the risk of BSI greatly [19]. Therefore, it is crucial to monitor the use of glucocorticoids rationally according to the condition [52].

### Incidence of ICU-BSI in COVID-19 patients

Meta-analysis unraveled that the incidence of BSI in COVID-19 patients in ICUs was 19.9%, similar to the currently reported 10–50% incidence rate. Compared with previous studies, in which 7% of COVID-19 hospitalized patients may experience BSI [2], the incidence of BSI in COVID-19 patients in ICUs increased nearly threefold. The incidence of ICU-BSI in COVID-19 patients varied in different studies, mainly because the occurrence of BSI lies in the detection of blood cultures. Also, it is somewhat difficult to exclude sampling contamination and detection contamination, and most patients in ICUs receive various types of medications, which may affect the detection of BSI [15, 71].

### Strengths and limitations

This is the first systematic review analyzing risk factors for ICU-BSI in COVID-19 patients, and the data were reviewed by two investigators to ensure accuracy. By incorporating an extensive array of papers with high quality, our findings provide an accurate and reliable framework for promptly identifying the risk of BSI occurrence in COVID-19 patients in ICUs. In addition, our findings provide more comprehensive references of risk factors for ICU-BSI in COVID-19 patients for clinical treatment, which is a guide for early prevention of BSI.

However, some limitations need discussion. First, the studies covered diverse ethnicities, populations, methods, and periods of investigation, which is reflected in heterogeneity. However, this may be due to differences in study design rather than actual differences in outcome measures. Therefore, we used sensitivity analyses and random-effects models to verify the result stability in the presence of high heterogeneity. Second, the diagnosis of BSI in COVID-19 patients treated with Tocilizumab may be difficult because patients often do not have fever and have low serum levels of typical inflammatory markers, requiring further study. Third, Data on diseases such as diabetes and oncology did not have specific types of data, so specific rich data are needed to study their association with BSI. Fourth, few of the included studies analyzed the impact of post-invasive treatment care measures on the occurrence of BSI in COVID-19 patients in ICUs. Therefore, in the future, more assessments of the impact of treatment details on ICU-BSI in COVID-19 patients and randomized controlled trials are needed to enhance the reliability.

## Conclusion

Our findings showed that males, higher SAPS II scores, DM, tracheal intubation, mechanical ventilation, ECMO, CVC, longer ICU stays, and Methylprednisolone use increased the risk of ICU-BSI in COVID-19 patients. It is imperative for future research to integrate these factors into a comprehensive predictive assessment framework to identify and intervene promptly in COVID-19 patients at high risk for ICU-BSI to improve treatment outcomes and promote patient health recovery.

## Supplementary Information

Supplementary Material 1.

## Data Availability

The datasets used during the current study are available from the corresponding author on reasonable request.
