# Supplementary material for: Risk factors for bloodstream infection in COVID-19 patients in intensive care units: a systematic review and meta-analysis
Source: BMC Infect Dis. 2025 Jan 3;25:13. doi: 10.1186/s12879-024-10420-1 (PMC11697680; doi:10.1186/s12879-024-10420-1)
Supplement: Supplementary file 1 — Supplementary Material 1. [file 12879_2024_10420_MOESM1_ESM.docx]

1.

（B）

（D）

（C）

Figure S1. Sensitivity analysis of univariate data associating BSI risk with (A)gender (B)diabetes (C)hypertension and (D) chronic pulmonary disease for patients with COVID-19 in ICU.

1.

Figure S2. Sensitivity analysis of univariate data associating BSI risk with (A)liver disease (B)chronic kidney disease (C)heart disease (D)immunosuppressive disease and (E)tumors for patients with COVID-19 in ICU.

（E）

（B）

（D）

（C）

（F）

（E）

Figure S3. Sensitivity analysis of univariate data aassociating BSI risk with (A)tracheal intubation(B)ECMO (C)Length of stay in ICU (D)Tocilizumab (E)Steroids and (F) Dexamethasone for patients with COVID-19 in ICU.

（D）

（C）

（A）

（B）

Table S1: Search strategy.

PubMed

| Search number | Query | Search Details | Results | Time |
| --- | --- | --- | --- | --- |
| 10 | #3 AND #6 AND #9 | ("Intensive Care Units"[MeSH Terms] OR ("intensive care unit*"[Title/Abstract] OR "ICU"[Title/Abstract] OR "critical*"[Title/Abstract] OR (("close"[All Fields] OR "closed"[All Fields] OR "closely"[All Fields] OR "closeness"[All Fields] OR "closes"[All Fields] OR "closing"[All Fields] OR "closings"[All Fields]) AND "attention unit"[Title/Abstract]) OR "intensive care*"[Title/Abstract] OR "intensive therapy unit"[Title/Abstract] OR "intensive treatment unit"[Title/Abstract] OR "special care unit"[Title/Abstract] OR "respiratory care unit*"[Title/Abstract])) AND ("COVID-19"[MeSH Terms] OR ("COVID-19"[Title/Abstract] OR "2019 ncov infection*"[Title/Abstract] OR "2019 novel coronavirus*"[Title/Abstract] OR "covid*"[Title/Abstract] OR "coronavirus disease*"[Title/Abstract] OR "severe acute respiratory syndrome coronavirus 2 infection"[Title/Abstract] OR "2019 ncov disease*"[Title/Abstract] OR "coronavirus disease 2019"[Title/Abstract] OR "coronavirus infection 2019"[Title/Abstract] OR "ncov 2019*"[Title/Abstract] OR "new coronavirus pneumonia"[Title/Abstract] OR "novel coronavirus*"[Title/Abstract] OR ("paucisymptomatic"[All Fields] AND "coronavirus disease 2019"[Title/Abstract]) OR "sars coronavirus 2*"[Title/Abstract] OR "sars cov 2*"[Title/Abstract] OR "sarscov2*"[Title/Abstract] OR "severe acute respiratory syndrome*"[Title/Abstract] OR "wuhan coronavirus*"[Title/Abstract])) AND ("Sepsis"[MeSH Terms] OR ("Sepsis"[Title/Abstract] OR "bloodstream infection*"[Title/Abstract] OR "pyemia*"[Title/Abstract] OR "pyohemia*"[Title/Abstract] OR "pyaemia*"[Title/Abstract] OR "septicemia*"[Title/Abstract] OR "blood poisoning*"[Title/Abstract] OR "blood stream infection*"[Title/Abstract] OR "blood borne infections"[Title/Abstract] OR "bacteremia*"[Title/Abstract])) | 1,529 | 10:22:48 |
| 9 | ("Sepsis"[Mesh]) OR ((((((((((Sepsis[Title/Abstract]) OR (Bloodstream Infection*[Title/Abstract])) OR (Pyemia*[Title/Abstract])) OR (Pyohemia*[Title/Abstract])) OR (Pyaemia*[Title/Abstract])) OR (Septicemia*[Title/Abstract])) OR (Blood Poisoning*[Title/Abstract])) OR (blood stream infection*[Title/Abstract])) OR (blood-borne Infections[Title/Abstract])) OR (Bacteremia*[Title/Abstract])) | "Sepsis"[MeSH Terms] OR "Sepsis"[Title/Abstract] OR "bloodstream infection*"[Title/Abstract] OR "pyemia*"[Title/Abstract] OR "pyohemia*"[Title/Abstract] OR "pyaemia*"[Title/Abstract] OR "septicemia*"[Title/Abstract] OR "blood poisoning*"[Title/Abstract] OR "blood stream infection*"[Title/Abstract] OR "blood borne infections"[Title/Abstract] OR "bacteremia*"[Title/Abstract] | 247,385 | 10:22:33 |
| 8 | (((((((((Sepsis[Title/Abstract]) OR (Bloodstream Infection*[Title/Abstract])) OR (Pyemia*[Title/Abstract])) OR (Pyohemia*[Title/Abstract])) OR (Pyaemia*[Title/Abstract])) OR (Septicemia*[Title/Abstract])) OR (Blood Poisoning*[Title/Abstract])) OR (blood stream infection*[Title/Abstract])) OR (blood-borne Infections[Title/Abstract])) OR(Bacteremia*[Title/Abstract]) | "Sepsis"[Title/Abstract] OR "bloodstream infection*"[Title/Abstract] OR "pyemia*"[Title/Abstract] OR "pyohemia*"[Title/Abstract] OR "pyaemia*"[Title/Abstract] OR "septicemia*"[Title/Abstract] OR "blood poisoning*"[Title/Abstract] OR "blood stream infection*"[Title/Abstract] OR "blood borne infections"[Title/Abstract] OR "bacteremia*"[Title/Abstract] | 181,572 | 10:22:18 |
| 7 | "Sepsis"[Mesh] | "Sepsis"[MeSH Terms] | 146,509 | 10:22:06 |
| 6 | ("COVID-19"[Mesh]) OR ((((((((((((((((((COVID-19[Title/Abstract]) OR (2019-nCoV Infection*[Title/Abstract])) OR (2019 Novel Coronavirus*[Title/Abstract])) OR (COVID*[Title/Abstract])) OR (Coronavirus Disease*[Title/Abstract])) OR (Severe Acute Respiratory Syndrome Coronavirus 2 Infection[Title/Abstract])) OR (2019-nCoV Disease*[Title/Abstract])) OR (coronavirus disease 2019[Title/Abstract])) OR (coronavirus infection 2019[Title/Abstract])) OR (nCoV 2019*[Title/Abstract])) OR (new coronavirus pneumonia[Title/Abstract])) OR (novel coronavirus*[Title/Abstract])) OR (paucisymptomatic coronavirus disease 2019[Title/Abstract])) OR (SARS coronavirus 2*[Title/Abstract])) OR (SARS-CoV-2*[Title/Abstract])) OR (SARSCoV2*[Title/Abstract])) OR (severe acute respiratory syndrome*[Title/Abstract])) OR (Wuhan coronavirus*[Title/Abstract])) | "COVID-19"[MeSH Terms] OR ("COVID-19"[Title/Abstract] OR "2019 ncov infection*"[Title/Abstract] OR "2019 novel coronavirus*"[Title/Abstract] OR "covid*"[Title/Abstract] OR "coronavirus disease*"[Title/Abstract] OR "severe acute respiratory syndrome coronavirus 2 infection"[Title/Abstract] OR "2019 ncov disease*"[Title/Abstract] OR "coronavirus disease 2019"[Title/Abstract] OR "coronavirus infection 2019"[Title/Abstract] OR "ncov 2019*"[Title/Abstract] OR "new coronavirus pneumonia"[Title/Abstract] OR "novel coronavirus*"[Title/Abstract] OR ("paucisymptomatic"[All Fields] AND "coronavirus disease 2019"[Title/Abstract]) OR "sars coronavirus 2*"[Title/Abstract] OR "sars cov 2*"[Title/Abstract] OR "sarscov2*"[Title/Abstract] OR "severe acute respiratory syndrome*"[Title/Abstract] OR "wuhan coronavirus*"[Title/Abstract]) | 435,710 | 10:21:51 |
| 5 | (((((((((((((((((COVID-19[Title/Abstract]) OR (2019-nCoV Infection*[Title/Abstract])) OR (2019 Novel Coronavirus*[Title/Abstract])) OR (COVID*[Title/Abstract])) OR (Coronavirus Disease*[Title/Abstract])) OR (Severe Acute Respiratory Syndrome Coronavirus 2 Infection[Title/Abstract])) OR (2019-nCoV Disease*[Title/Abstract])) OR (coronavirus disease 2019[Title/Abstract])) OR (coronavirus infection 2019[Title/Abstract])) OR (nCoV 2019*[Title/Abstract])) OR (new coronavirus pneumonia[Title/Abstract])) OR (novel coronavirus*[Title/Abstract])) OR (paucisymptomatic coronavirus disease 2019[Title/Abstract])) OR (SARS coronavirus 2*[Title/Abstract])) OR (SARS-CoV-2*[Title/Abstract])) OR (SARSCoV2*[Title/Abstract])) OR (severe acute respiratory syndrome*[Title/Abstract])) OR (Wuhan coronavirus*[Title/Abstract]) | "COVID-19"[Title/Abstract] OR "2019 ncov infection*"[Title/Abstract] OR "2019 novel coronavirus*"[Title/Abstract] OR "covid*"[Title/Abstract] OR "coronavirus disease*"[Title/Abstract] OR "severe acute respiratory syndrome coronavirus 2 infection"[Title/Abstract] OR "2019 ncov disease*"[Title/Abstract] OR "coronavirus disease 2019"[Title/Abstract] OR "coronavirus infection 2019"[Title/Abstract] OR "ncov 2019*"[Title/Abstract] OR "new coronavirus pneumonia"[Title/Abstract] OR "novel coronavirus*"[Title/Abstract] OR ("paucisymptomatic"[All Fields] AND "coronavirus disease 2019"[Title/Abstract]) OR "sars coronavirus 2*"[Title/Abstract] OR "sars cov 2*"[Title/Abstract] OR "sarscov2*"[Title/Abstract] OR "severe acute respiratory syndrome*"[Title/Abstract] OR "wuhan coronavirus*"[Title/Abstract] | 427,924 | 10:21:31 |
| 4 | "COVID-19"[Mesh] | "COVID-19"[MeSH Terms] | 268,269 | 10:21:10 |
| 3 | ("Intensive Care Units"[Mesh]) OR (((((((((Intensive Care Unit*[Title/Abstract]) OR (ICU[Title/Abstract])) OR (Critical*[Title/Abstract])) OR (close attention unit[Title/Abstract])) OR (intensive care*[Title/Abstract])) OR (intensive therapy unit[Title/Abstract])) OR (intensive treatment unit[Title/Abstract])) OR (special care unit[Title/Abstract])) OR (respiratory care unit*[Title/Abstract])) | "Intensive Care Units"[MeSH Terms] OR ("intensive care unit*"[Title/Abstract] OR "ICU"[Title/Abstract] OR "critical*"[Title/Abstract] OR (("close"[All Fields] OR "closed"[All Fields] OR "closely"[All Fields] OR "closeness"[All Fields] OR "closes"[All Fields] OR "closing"[All Fields] OR "closings"[All Fields]) AND "attention unit"[Title/Abstract]) OR "intensive care*"[Title/Abstract] OR "intensive therapy unit"[Title/Abstract] OR "intensive treatment unit"[Title/Abstract] OR "special care unit"[Title/Abstract] OR "respiratory care unit*"[Title/Abstract]) | 1,363,872 | 10:20:46 |
| 2 | ((((((((Intensive Care Unit*[Title/Abstract]) OR (ICU[Title/Abstract])) OR (Critical*[Title/Abstract])) OR (close attention unit[Title/Abstract])) OR (intensive care*[Title/Abstract])) OR (intensive therapy unit[Title/Abstract])) OR (intensive treatment unit[Title/Abstract])) OR (special care unit[Title/Abstract])) OR (respiratory care unit*[Title/Abstract]) | "intensive care unit*"[Title/Abstract] OR "ICU"[Title/Abstract] OR "critical*"[Title/Abstract] OR (("close"[All Fields] OR "closed"[All Fields] OR "closely"[All Fields] OR "closeness"[All Fields] OR "closes"[All Fields] OR "closing"[All Fields] OR "closings"[All Fields]) AND "attention unit"[Title/Abstract]) OR "intensive care*"[Title/Abstract] OR "intensive therapy unit"[Title/Abstract] OR "intensive treatment unit"[Title/Abstract] OR "special care unit"[Title/Abstract] OR "respiratory care unit*"[Title/Abstract] | 1,338,070 | 10:20:17 |
| 1 | "Intensive Care Units"[MeSH Terms] | "Intensive Care Units"[MeSH Terms] | 110,653 | 10:19:41 |

Embase

| No. | Query | Results | Date |
| --- | --- | --- | --- |
| #10 | #3 AND #6 AND #9 | 3197 | 21-Jul-24 |
| #9 | #7 OR #8 | 346889 | 21-Jul-24 |
| #8 | sepsis:ab,ti OR 'bloodstream infection*':ab,ti OR pyemia*:ab,ti OR pyohemia*:ab,ti OR pyaemia*:ab,ti OR septicemia*:ab,ti OR 'blood poisoning*':ab,ti OR 'blood stream infection*':ab,ti OR 'blood-borne infections':ab,ti OR 'bacteremia*':ab,ti | 267798 | 21-Jul-24 |
| #7 | 'bloodstream infection'/exp | 140348 | 21-Jul-24 |
| #6 | #4 OR #5 | 523881 | 21-Jul-24 |
| #5 | 'covid 19':ab,ti OR '2019-ncov infection*':ab,ti OR '2019 novel coronavirus*':ab,ti OR covid*:ab,ti OR 'coronavirus disease*':ab,ti OR 'severe acute respiratory syndrome coronavirus 2 infection':ab,ti OR '2019-ncov disease*':ab,ti OR 'coronavirus disease 2019':ab,ti OR 'coronavirus infection 2019':ab,ti OR 'ncov 2019*':ab,ti OR 'new coronavirus pneumonia':ab,ti OR 'novel coronavirus*':ab,ti OR 'paucisymptomatic coronavirus disease 2019':ab,ti OR 'sars coronavirus 2*':ab,ti OR 'sars cov 2*':ab,ti OR sarscov2*:ab,ti OR 'severe acute respiratory syndrome*':ab,ti OR 'wuhan coronavirus*':ab,ti | 484267 | 21-Jul-24 |
| #4 | 'coronavirus disease 2019'/exp | 411334 | 21-Jul-24 |
| #3 | #1 OR #2 | 1828132 | 21-Jul-24 |
| #2 | 'intensive care units':ab,ti OR 'intensive care unit':ab,ti OR icu:ab,ti OR critical*:ab,ti OR 'close attention unit':ab,ti OR 'intensive care*':ab,ti OR 'intensive therapy unit':ab,ti OR 'intensive treatment unit':ab,ti OR 'special care unit':ab,ti OR 'respiratory care unit*':ab,ti | 1727228 | 21-Jul-24 |
| #1 | 'intensive care unit'/exp | 324001 | 21-Jul-24 |

Web of science

| Entitlements | # | Search Query | Database | Results | Date Run |
| --- | --- | --- | --- | --- | --- |
| - WOS: 1985 to 2024 - CSCD: 1989 to 2024 - KJD: 1980 to 2024 - MEDLINE: 1950 to 2024 - PPRN: 1991 to 2024 - PQDT: 1637 to 2024 - SCIELO: 2002 to 2024 | 1 | TS=(Intensive Care Units or Intensive Care Unit or ICU or Critical* or close attention unit or intensive care* or intensive therapy unit or intensive treatment unit or special care unit or respiratory care unit*) and Preprint Citation Index (Exclude – Database) | All Databases | 3033364 | Wed Jul 10 2024 18:52:48 GMT+0800 |
| - WOS: 1985 to 2024 - CSCD: 1989 to 2024 - KJD: 1980 to 2024 - MEDLINE: 1950 to 2024 - PPRN: 1991 to 2024 - PQDT: 1637 to 2024 - SCIELO: 2002 to 2024 | 2 | TS=(COVID 19 or 2019 nCoV Infection* or 2019 Novel Coronavirus* or COVID* or Coronavirus Disease* or Severe Acute Respiratory Syndrome Coronavirus 2 Infection or 2019 nCoV Disease* or coronavirus disease 2019 or coronavirus infection 2019 or nCoV 2019* or new coronavirus pneumonia or novel coronavirus* or paucisyptomatic coronavirus disease 2019 or SARS coronavirus 2 or SARS CoV 2 or SARSCoV2* or severe acute respiratory syndrome* or Wuhan coronavirus* ) and Preprint Citation Index (Exclude – Database) | All Databases | 654724 | Wed Jul 10 2024 18:54:42 GMT+0800 |
| - WOS: 1985 to 2024 - CSCD: 1989 to 2024 - KJD: 1980 to 2024 - MEDLINE: 1950 to 2024 - PPRN: 1991 to 2024 - PQDT: 1637 to 2024 - SCIELO: 2002 to 2024 | 3 | TS=(Sepsis or Bloodstream Infection* or Pyemia* or Pyohemia* or Pyaemia* or Septicemia* or Blood Poisoning* or blood stream infection* or blood-borne Infections or bacteremia ) and Preprint Citation Index (Exclude – Database) | All Databases | 452530 | Wed Jul 10 2024 18:55:46 GMT+0800 |
| - WOS: 1985 to 2024 - CSCD: 1989 to 2024 - KJD: 1980 to 2024 - MEDLINE: 1950 to 2024 - PPRN: 1991 to 2024 - PQDT: 1637 to 2024 - SCIELO: 2002 to 2024 | 4 | #1 and #2 and #3 and Preprint Citation Index (Exclude – Database) | All Databases | 4067 | Wed Jul 10 2024 18:56:11 GMT+0800 |

Cochrane library

| ID | Search |
| --- | --- |
| #1 | MeSH descriptor: [Intensive Care Units] explode all trees |
| #2 | (Intensive Care Units or Intensive Care Unit or ICU or Critical* or close attention unit or intensive care* or intensive therapy unit or intensive treatment unit or special care unit or respiratory care unit*):ti,ab,kw |
| #3 | #1 or #2 |
| #4 | MeSH descriptor: [COVID-19] explode all trees |
| #5 | (COVID 19 or 2019 nCoV Infection* or 2019 Novel Coronavirus* or COVID* or Coronavirus Disease* or Severe Acute Respiratory Syndrome Coronavirus 2 Infection or 2019 nCoV Disease* or coronavirus disease 2019 or coronavirus infection 2019 or nCoV 2019* or new coronavirus pneumonia or novel coronavirus* or paucisymptomatic coronavirus disease 2019 or SARS coronavirus 2* or SARS CoV 2* or SARSCoV2* or severe acute respiratory syndrome* or Wuhan coronavirus*):ti,ab,kw |
| #6 | #4 or #5 |
| #7 | MeSH descriptor: [Sepsis] explode all trees |
| #8 | (Sepsis or Bloodstream Infection* or Pyemia* or Pyohemia* or Pyaemia* or Septicemia* or Blood Poisoning* or blood stream infection* or blood-borne Infections or bacteremia): ti,ab,kw |
| #9 | #7 or #8 |
| #10 | #3 and #6 and #9 |
